# Dieulafoy Lesion of the Colon: A Rare Finding During Colonoscopy

**DOI:** 10.7759/cureus.25188

**Published:** 2022-05-21

**Authors:** Sohaib Khan, Saleha Niaz, Rajiv Singh, Stephanie Lucas, Christopher Calcagno

**Affiliations:** 1 Internal Medicine, Parkview Medical Center, Pueblo, USA; 2 Medicine, Nawaz Sharif Medical College, Gujrat, PAK; 3 Gastroenterology, Parkview Medical Center, Pueblo, USA

**Keywords:** angiographic embolization, endo clipping, thermal coagulation, epinephrine injection, obscure bleeding, erosion, lower gastrointestinal bleeding, colonoscopy, mucosal ulceration, dieulafoy lesion

## Abstract

Dieulafoy lesions are common dilated submucosal vessels that can present with gastrointestinal (GI) bleeding. These lesions are usually found in the stomach or esophagus and colonic Dieulafoy lesions are very rare. Clinical presentation can vary from mild non-threatening GI bleeding to massive and recurrent hemorrhage. Here, we discuss a case of a 71-year-old female patient who presented with a bright red bleed per rectum. Colonoscopy was performed, which revealed a bleeding Dieulafoy lesion in the descending colon with clotted blood in the transverse and descending colon. In this article, we will also review the literature related to the epidemiology, clinical presentation, diagnosis, and management of Dieulafoy lesions.

## Introduction

A Dieulafoy lesion is defined as an aberrant dilated submucosal artery that erodes the overlying mucosal barrier without any mucosal ulceration [1,2]. First explained by Gallard in 1884 and named by a French surgeon Dieulafoy in 1898, they are responsible for 1-2% of acute gastrointestinal (GI) bleeding and can cause significant morbidity and mortality [1-4]. In the GI tract, they are mostly found in the stomach (70%) and the esophagus (15%). With only 2% prevalence in the colon, Dieulafoy lesions are a very rare cause of lower GI bleeding as compared to more common causes like hemorrhoids, angiodysplasia, and diverticulosis [1-3]. Due to their small size and caliber, they are very difficult to diagnose endoscopically and can pose a diagnostic challenge [5]. Here, we present a case of a 71-year-old patient, who was found to have significant lower GI bleeding secondary to a Dieulafoy lesion in the descending colon.

## Case presentation

**Figure 1:**
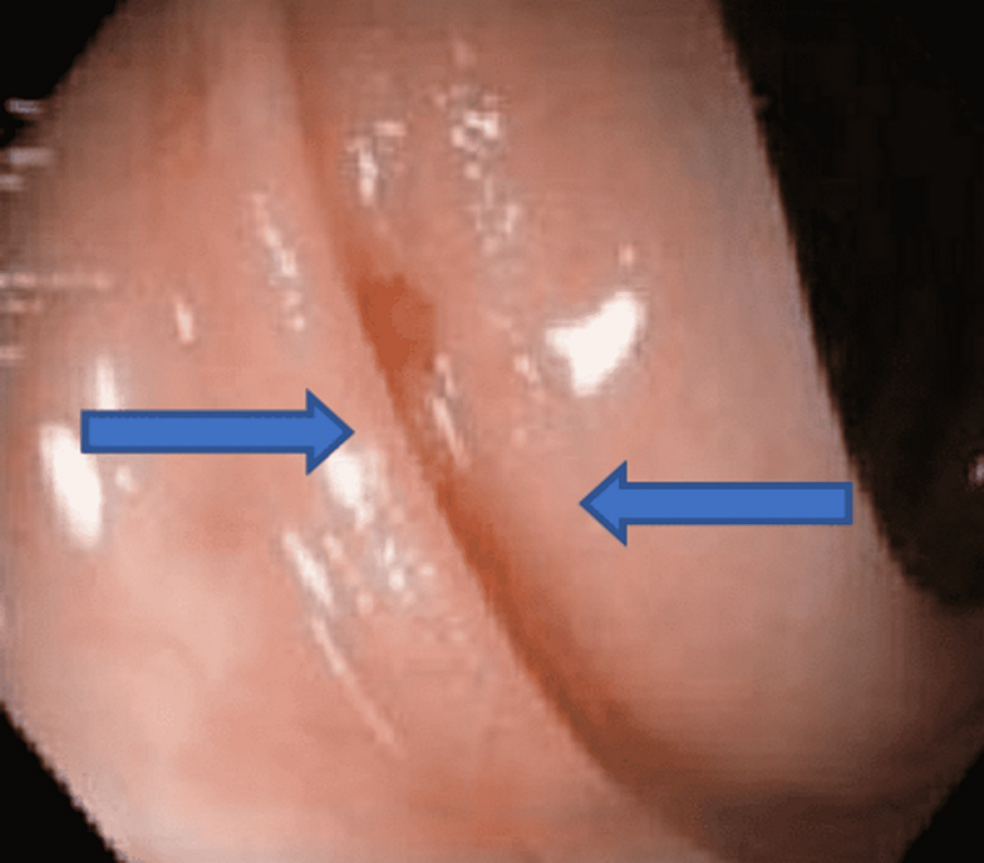
Dieulafoy lesion (blue arrows) in the descending colon.

**Figure 2:**
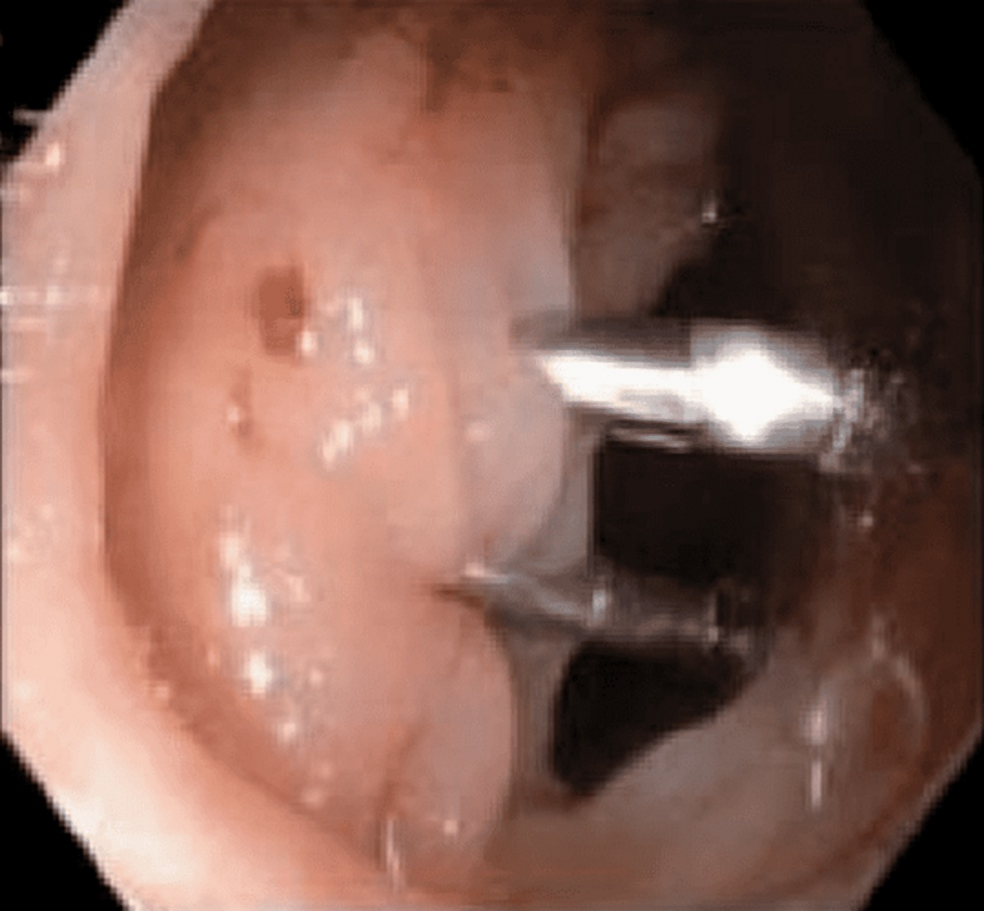
Two endoclips applied for the purpose of hemostasis.

A 71-year-old female patient was admitted to the hospital due to concerns of rectal bleeding. Her symptoms started two days prior with bright red blood per rectum (BRBPR) and were associated with generalized abdominal pain. Vitals upon presentation were within normal limits. Labs were significant for a hemoglobin level of 4.5 g/dl, hematocrit of 15.7%, mean corpuscular volume (MCV) of 95.2 fL, platelet count of 202/µL, international normalized ratio (INR) of 3.2, prothrombin time (PT) of 33.6 seconds, blood urea nitrogen (BUN) of 25 mmol/L, creatinine of 1.06 mg/dL, and glomerular filtration rate (GFR) of 51 ml/min. A computed tomography (CT) scan of the abdomen and pelvis did not show any acute intra-abdominal pathology. Due to hemodynamic instability from lower GI bleeding, two large-bore intravenous (IV) lines were placed, and the patient was started on IV fluids and IV pantoprazole infusion. Due to significantly low hemoglobin and supra-therapeutic INR, Coumadin was held, and the patient was transfused three units of packed red blood cells (PRBC) and two units of fresh frozen plasma (FFP). The patient subsequently underwent colonoscopy, which showed a bleeding Dieulafoy lesion in the descending colon along with clotted blood in the distal rectum, descending colon, and distal transverse colon (Figure 1). Hemostasis was achieved with two endoclips that were applied to the lesion in the descending colon (Figure 2). No other source of bleeding was found during the colonoscopy. After the procedure, the patient did not have any recurrent bleeding and hemoglobin/hematocrit remained stable. Warfarin was stopped as per recommendations from vascular surgery. Once hemodynamically stable, the patient was discharged with follow-up in the gastroenterology and vascular surgery outpatient clinics.

## Discussion

Colonic Dieulafoy lesions are a rare cause of lower GI bleeding but can be responsible for massive and recurrent bleeding if left untreated [6]. Our case with a Dieulafoy lesion in the descending colon makes for a rare presentation, as the major predominance of these lesions is in the stomach and esophagus [3]. The proposed pathophysiologic mechanism of a Dieulafoy lesion includes microtrauma and ischemia of overlying mucosa, subsequently leading to thinning of the mucosal wall, making it highly vulnerable to erosion and hemorrhage [7]. Clinical presentation varies from mild bleeding episodes to massive hemorrhage, with an increased risk with comorbidities like hypertension, cardiovascular disease, and chronic kidney disease with or without anticoagulation use [1]. Constipation or cancer also poses a significant risk for colonic Dieulafoy lesion due to mechanical factors [7].

A Dieulafoy lesion is usually small in size with intermittent bleeding; hence, making diagnosis particularly challenging on colonoscopy. Poor bowel preparation, obscure bleeding, and potential concomitant diverticula contribute to potential underdiagnoses of this clinical entity [2]. Hence, both endoscopic and angiographic modalities can be used for diagnosis [8].

Various treatment approaches have been used for bleeding hemostasis, including epinephrine injection, sclerotherapy, thermal coagulation, and mechanical therapy with band ligation or endoclipping [4]. Angiographic embolization can also be used for refractory cases while surgical resection is reserved only as a last resort [4]. Endoscopic clipping has been considered ideal for colonic Dieulafoy lesions with better hemostatic effects, reduced risk of rebleeding, and decreased complication risk [9,10]. Our patient achieved hemostasis without any subsequent rebleeding after endoclip placement in the descending colon.

## Conclusions

Colonic Dieulafoy lesions must be considered in the differential diagnosis for lower GI bleeding as they can cause obscure bleeding. Our case describes lower GI bleeding due to the rare presence of a Dieulafoy lesion in the descending colon. Better localization requires adequate bowel preparation and high clinical suspicion. Sustained hemostasis can be achieved with endoscopic treatments including epinephrine injection, sclerotherapy, band ligation, or endoclipping. Angiographic embolization and eventually surgical resection can be used for refractory cases.
